# Scurvy: Forgotten diagnosis, but still exist

**DOI:** 10.1016/j.ijscr.2020.03.002

**Published:** 2020-03-07

**Authors:** Faisal Miraj, Ali Abdullah

**Affiliations:** a Department of Orthopaedic – Traumatology, Fatmawati General Hospital, Indonesia; b Department of Orthopaedic – Traumatology, Faculty of Medicine Universitas, Indonesia

**Keywords:** Scurvy, Clinical and radiological appearance, Vitamin C administration

## Abstract

**Introduction:**

Scurvy is a rare condition in pediatric patients, resulted from a vitamin C deficiency. Musculoskeletal symptoms occur in 80% of patient. The rarity and the polymorphisms of the clinical signs and symptoms often make scurvy an unknown or forgotten diagnosis.

**Methods:**

A 3-years-old boy with limping gait, progressive pain and swelling on his left lower limb since 3 months prior was diagnosed as osteomyelitis based on MRI. When there was no improvement after 2 weeks of IV antibiotic administration, we did surgical debridement procedure. Intraoperative, we found extensive blood clot inside and in the entire periosteum instead of infection. One week after surgery, there was still no improvement clinically and radiographically. Histopathology result suggested a fibro-osseous tumor. After reviewing clinical sign including skin presentation and plain radiology, we suspected scurvy disease in this patient and started a vitamin C therapy course immediately.

**Results:**

Two weeks after we started vitamin C treatment, the patient’s condition was significantly improved clinically and radiographically. Serial radiograph was taken after 2 and 12 months of surgery, and it showed significant improvement on the site where the previous pathological process appeared.

**Discussions:**

Because of the extremely rare occurrence of scurvy in modern society at present, it is difficult to differentiate it from other diseases such as infection. The diagnosis of scurvy is made by careful studies from history taking, physical examination, laboratory and especially radiological finding that show pathognomonic appearance. Examination of vitamin C level serum is a gold standard, but unfortunately not available in our country. Improvement after vitamin C administration also established that condition.

**Conclusion:**

Scurvy should have established by clinical and radiological findings that show pathognomonic signs around joint, even without supported by vitamin C serum.

## Highlights

• Scurvy is a rare condition in pediatric patients.
• The rarity and the polymorphisms of the clinical signs and symptoms make scurvy an often unknown or forgotten diagnosis.
• Extremely rare occurrence of scurvy in modern society at present time, it is difficult to differentiate it from other diseases such as infection.

## 1 Introduction

Scurvy is a rare condition in pediatric patients, resulted from a deficiency of vitamin C (ascorbic acid). A marked reduction in the occurrence of scurvy has occurred over the last 100 years due to improved knowledge about the pathophysiology and treatment of scurvy. However, the disease still exists even in industrialized countries [1,2].

Musculoskeletal symptoms are prominent in pediatric scurvy and occur in 80% of patients [3,4]. We can find non-specific arthralgia, myalgia, hemarthrosis, muscular hemorrhage, and subperiosteal hematomas. These symptoms are more common in the lower extremities and the knee is the most affected joint [3]. The rarity and the polymorphisms of the clinical signs and symptoms make scurvy an often unknown or forgotten diagnosis.

We reported a case of scurvy in a 3-years-old boy, based upon clinical presentation, radiography, and magnetic resonance imaging led to a surgery for debridement of an osteomyelitis with negative finding rather than revealing subperiosteal and intramedullary hematomas. The case has been reported in line with the SCARE criteria for better understanding [5].

## 2 Case presentation

A 3-years-old boy came to our hospital with pain and swelling on his left lower limb. He started walking with a limping gait 3 months before. His parents took him to a pediatrician and an orthopedic surgeon. They were told that there was no problem with his musculoskeletal system. He was suggested to undergo physiotherapy to relieve the pain.

After a few weeks, the pain persisted and the swelling was worsened. He tended no move his left leg. He was also presented with general weakness, poor oral intake and mild fever for two weeks long. He was admitted to our hospital and underwent several radiographic and laboratory examinations.

Upon physical examination, the patient’s weight was below the third percentile for his age group. The patient’s left leg was swollen and warm, but there was no erythema. We also revealed corkscrews hair. The laboratory data results were as follows: white blood cell count 5.200 K/ul; neutrophil count 37.4%; lymphocyte count 53.7%; and platelet count 381.000 K/ul. Furthermore, the erythrocyte sedimentation rate was 15 mm/hr, the C-reactive protein level was <5 mg/dL, and the hemoglobin (9.2 g/dL) was slightly decreased. Hemostasis, alkaline phosphatase, calcium, and phosphate levels were normal.

**Fig. 1:**
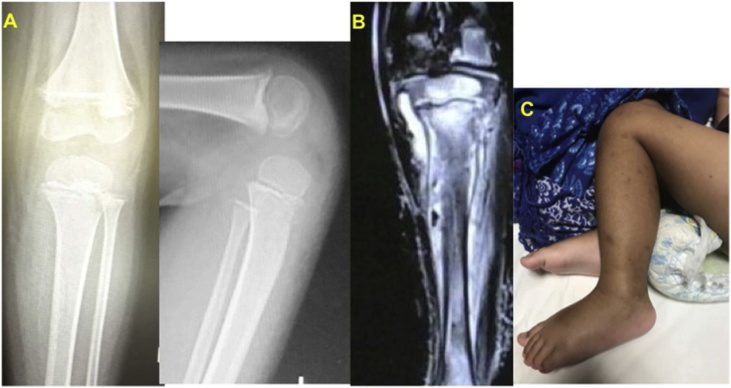
(A). Plain radiology findings: signs of osteopenia, the thick sclerotic metaphyseal line above a widened physis, and small beak-like excrescences at the metaphysis of both tibias, (B). High signal intensity and fluid-fluid levels on T2-weighted images on the proximal tibia. Based on MRI examination we suggest an osteomyelitis, (C). Clinical sign: swelling, unwilling to bear weight.

The radiographs of both knees showed signs of osteopenia, the thick sclerotic metaphyseal line above a widened physis, and small beak-like excrescences at the metaphysis of both tibias. We could also see a radiolucent shadow at proximal physis of the tibia and periosteal elevation (Fig. 1).

An MRI of the left leg performed and revealed diffuse bone marrow signal changes in the proximal shaft of the tibia with subperiosteal fluid collection and displacement of the proximal epiphysis. The marrow changes appeared as heterogeneous high and low signal intensities on T1-weighted images and heterogeneous high and intermediate signal intensity on T2-weighted images. The subperiosteal fluid collection had low signal intensity on T1-weighted images and high signal intensity and fluid-fluid levels on T2-weighted images. The periosteum and surrounding muscles of the leg were moderately enhanced on contrast-enhanced fat-suppressed T1-weighted images (Fig. 1).

Based on the previous history, physical examination and radiographic findings especially MRI result, the previous pediatrician made a conclusion of osteomyelitis in the left tibia for the working diagnosis. So they began empiric antibiotic therapy. After 2 weeks of IV antibiotic administration, the symptoms were not improved. His left leg was getting more swollen. Pain and fever were persisted.

**Fig. 2:**
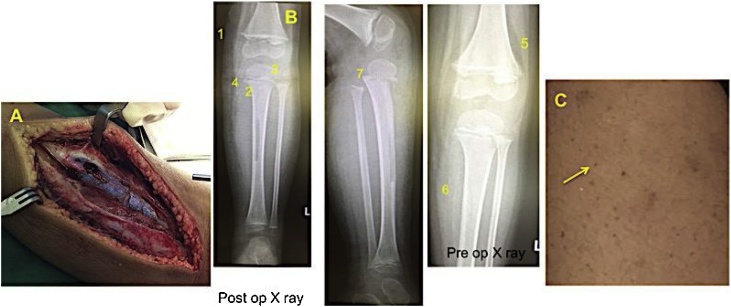
(A). Intraoperative findings; subperiosteal hematom; new bone formation; no pus; no cortical destruction; intramedullary hematoma, (B). One week post operative plain radiology finding: 1. Frankel Line; 2. Trummerfild zone (Scorbutic zone); 3. Ring epiphysis (Wimberger); 4. Pelkin spur; 5/6. Periosteal elevation (subperiosteal hematom) with new bone formation; 7. Epiphyseal slidesexamination, and (C). Corkscrew hairs.

Afterward a surgical procedure was performed to drain and irrigate the periosteal fluid collection in the left proximal tibia. A relatively healthy color and consistency were noted in the soft tissue along the incision tract. The dark serous hematoma was drained from the periosteal incision site and a mild chronic inflammation with hemorrhage inside the entire the periosteum. No sign of infection nor cortical destruction, A window was made over the cortex of proximal part followed by intramedullary cavity curettage and only revealed blood clot. (Fig. 2). We took a tissue sample and sent it to the pathology.

Two weeks after surgical debridement and antibiotics administration there was still not much improvement (Fig. 2). He was still presented with fever, malaise, anorexia, pale, and pain was also persisted. His left leg was still swollen and tender, but the surgical wound was already healed. He also resisted moving his leg.

The histopathology evaluation showed spindle to oval-shaped cells with fine chromatin, some of them with small nucleoli, eosinophilic-myxoid stroma, blood vessels with narrow lumen, and chronic inflammatory cells. The conclusion of histopathology evaluation was the fibro-osseous tumors appearance group.

Even though the histopathology suggested fibro-osseous tumors, the clinical and radiological findings were not consistent with it. Two weeks post-surgical radiograph examination was taken and showed a worsened bone destruction appearance on the proximal metaphyseal region of the tibia and pathognomonic sign that suspected to Scurvy disease. It was also consistent with patients history of pain and swollen limb, unwilling to bear weight, subfebris fever, malaise and corkscrew hairs along the lower leg.

From the Clinicopathological Conference forum, further histopathology review showed signs of recent subperiosteal bleeding, mineralized cartilaginous matrix of the trabecular and myelofibrosis, which was suggestive of Scurvy disease. Vitamin C serum level examination should be done for the diagnosis. But it was not available in our country.

So we began vitamin C therapy 300 mg per day divided into 2 times for 2 weeks. Then it was continued with 100 mg a day until the disease was resolved (1–3 months). After that, we gave him 30 mg a day as a maintenance dose. Two weeks after we started him on vitamin C treatment, the pain has significantly subsided, swelling on the leg and ankle was also decreased. He started to move his knee and ankle joint and regained his ability to walk. His appetite slowly came back.

**Fig. 3:**
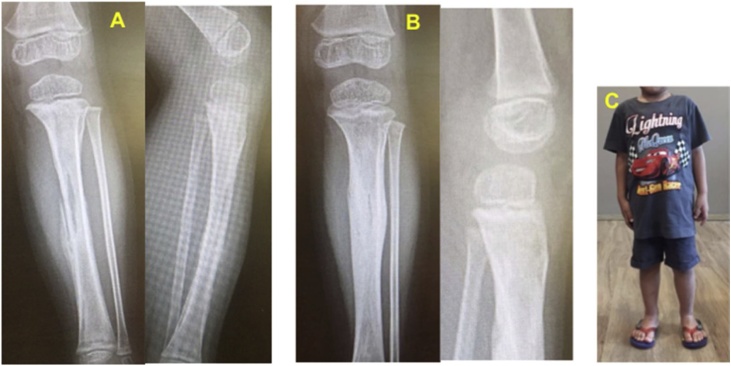
(A). Two months after high dose vitamin C treatment, (B) One year after high dose vitamin C treatment, (C). Painless and normal ambulation.

Two months with maintenance of vitamin C, radiograph showed significant improvement on the site of where the previous pathological process appeared. The metaphyseal region was sclerotic. The cortex was thickened. The bone defect was filled with numerous new bone formations. Clinically, the patient looked completely healthy and already came back into his previous daily activities. Those improvements persisted after one year followed up (Fig. 3).

## 3 Discussion

Vitamin C is essential for the human's body and much concerned in the maintenance of intercellular connective tissue and stabilization of collagen triple helix [6,7]. Since the human body is unable to synthesize vitamin C, its dietary intake must be in sufficient amounts [7]. Although it is rare, scurvy in pediatric patients is still found. Scurvy is frequently seen in babies whose fed with pasteurized milk because of the process that denatures the ascorbate [3,8].

Musculoskeletal manifestations of scurvy are found in 80% of scorbutic cases; the symptoms of these cases include arthralgia, myalgia, hemarthrosis, and muscular hematomas [2]. These symptoms are thought to be prominent in pediatric scurvy. In this case, pain and swollen of the patient’s leg made him walk with limping gait, limited range of motion and unwillingness to stand or walk. Prodromal symptoms persistence such as fever, malaise, anorexia, and corkscrew hairs.

The diagnosis of scurvy is mainly based on clinical presentation, plain radiograph findings of long bones and related history of poor intake of vitamin C, which was consistent since the patient does not like to have vegetables nor fruits. Radiographic findings which indicating scurvy are Frankel sign, Wimberger ring, and Corner sign (Pelkin spur). The Frankle sign is a white line at the ends of metaphyses. The Wimberger ring is a white line of calcification surrounding the epiphyseal centers. The Wimberger ring around the knee joint is one of the dominant radiograph findings of scurvy; a “ground-glass’’ appearance of the shaft; Scurvy lines: transverse bands of diminished density next to the Frankel sign; Corner sign (Pelkin spur): lateral metaphyseal spurs secondary to infarctions [8,9]. In this case Frankel line, scorbutic zone, Wimberger ring, Pelkin spur, periosteal elevation, and epiphyseal slides showed in radiology examination. But unfortunately those patognomonic radiological appearances noticed just after surgical debridement procedure (Fig. 2).

Skeletal changes in scurvy are most severe in young children because their bones are growing, and the periosteum is not as tightly bound to the surface of the cortex compared with adults [10,11]. Besides bone marrow lesions, bleeding within the joints, mainly in the hips, knees, and ankles may occur, because of damage to synovial blood vessels [2], but bleeding within the joints was not revealed on MRI in the present case.

MRI findings of the present case included diffuse bone marrow signal changes, a periosteal lesion, and diffuse muscular signal abnormalities in the leg suggested an osteomyelitis that directed to surgical debridement procedure. There are only a few reports regarding MRI findings of scurvy in children, such as diffuse heterogeneous signal intensity in the tibia shaft, and subperiosteal fluid/ hematoma with rim enhancement and surrounding soft tissue edema [10,12]. Actually these findings are generally consistent with the present case.

As MRI findings of the present case were not specific, pathological findings should be the key to correct diagnosis. There have been few recent studies that have focused on histopathological alterations in the tissue of patients with scurvy, probably because they are usually nonspecific. In the present case, although the biopsy specimen revealed the proliferation of young fibroblasts as well as several extravasated red cells and hemosiderin-laden histiocytes, fibrosis was minimal. This unusual histopathology implied disturbance of collagen formation, and the combined clinicopathological findings led to the diagnosis of scurvy.

The diagnosis of scurvy is sufficiently made by careful studies of history taking and physical examination, laboratory and especially radiological finding that shows pathognomonic appearance. Examination of vitamin C level serum is a gold standard, but unfortunately not available in our country. A low plasma level of vitamin C (<0.2 mg/dl) is specific in scurvy. Measuring the vitamin C level in the buffy-coat of the leucocytes is a better estimate of the vitamin body stores [6]. Immediate improvement of patient’s condition after vitamin C administration also establish that condition

## 4 Conclusion

Because of the extremely rare occurrence of scurvy in modern society at present, it is difficult to differentiate it from other diseases such as infection. Scurvy should have established by clinical and radiological findings that shows pathognomonic appearance around joint, even without supported by vitamin C serum.

Clinicians and radiologists must be aware of this extremely rare but still present condition because it is potentially fatal but easily managed with vitamin C supplementation.

## Declaration of Competing Interest

The authors have no ethical conflicts to disclose.

## Sources of funding

There is no sources of funding sponsor in this manuscript.

## Ethical approval

The authors have no ethical conflicts to disclose.

## Consent

Written informed consent was obtained from the patient's parents for publication of this case report and accompanying images. A copy of the written consent is available for review by the Editor-in-Chief of this journal on request.

## Author contribution

1. Faisal Mi’raj, MD. Contributed as making the case report, funding, and final approval of manuscript.
2. Ali Abdullah, MD. Contributed as making case report, collecting the data, and writing manuscript.

## Registration of research studies

N/A.

## Guarantor

Faisal Mi’raj, MD.

## Provenance and peer review

Not commissioned, externally peer-reviewed.
